# Resveratrol attenuates ICAM-1 expression and monocyte adhesiveness to TNF-α-treated endothelial cells: evidence for an anti-inflammatory cascade mediated by the miR-221/222/AMPK/p38/NF-κB pathway

**DOI:** 10.1038/srep44689

**Published:** 2017-03-24

**Authors:** Chen-Wei Liu, Hsin-Ching Sung, Shu-Rung Lin, Chun-Wei Wu, Chiang-Wen Lee, I.-Ta Lee, Yi-Fan Yang, I-Shing Yu, Shu-Wha Lin, Ming-Hsien Chiang, Chan-Jung Liang, Yuh-Lien Chen

**Affiliations:** 1 Department of Anatomy and Cell Biology, College of Medicine, National Taiwan University, Taipei, Taiwan; 2 Department of Anatomy, College of Medicine, Chang Gung University, Taoyuan, Taiwan; 3 Department of Bioscience Technology, College of Science, Chung-Yuan Christian University, Taoyuan, Taiwan; 4 Center for Nanotechnology and Center for Biomedical Technology, Chung-Yuan Christian University, Taoyuan, Taiwan; 5 Department of Nursing, Division of Basic Medical Sciences, and Chronic Diseases and Health Promotion Research Center, Chang Gung University of Science and Technology, Chia-Yi, Taiwan; 6 School of Medicine, College of Medicine, China Medical University, Taichung, Taiwan; 7 Department of Internal Medicine, National Taiwan University Hospital, Taipei, Taiwan; 8 Laboratory Animal Center, College of Medicine, National Taiwan University, Taipei, Taiwan; 9 Department of Clinical Laboratory Sciences and Medical Biotechnology, College of Medicine, National Taiwan University, Taipei, Taiwan; 10 Lipid Science and Aging Research Center, Kaohsiung Medical University, Kaohsiung, Taiwan; 11 Center for Lipid Biosciences, Kaohsiung Medical University Hospital, Taiwan.

## Abstract

Resveratrol, an edible polyphenolic phytoalexin, improves endothelial dysfunction and attenuates inflammation. However, the mechanisms have not been thoroughly elucidated. Therefore, we investigated the molecular basis of the effects of resveratrol on TNF-α-induced ICAM-1 expression in HUVECs. The resveratrol treatment significantly attenuated the TNF-α-induced ICAM-1 expression. The inhibition of p38 phosphorylation mediated the reduction in ICAM-1 expression caused by resveratrol. Resveratrol also decreased TNF-α-induced IκB phosphorylation and the phosphorylation, acetylation, and translocation of NF-κB p65. Moreover, resveratrol induced the AMPK phosphorylation and the SIRT1 expression in TNF-α-treated HUVECs. Furthermore, TNF-α significantly suppressed miR-221/-222 expression, which was reversed by resveratrol. miR-221/-222 overexpression decreased p38/NF-κB and ICAM-1 expression, which resulted in reduced monocyte adhesion to TNF-α-treated ECs. In a mouse model of acute TNF-α-induced inflammation, resveratrol effectively attenuated ICAM-1 expression in the aortic ECs of TNF-α-treated wild-type mice. These beneficial effects of resveratrol were lost in miR-221/222 knockout mice. Our data showed that resveratrol counteracted the TNF-α-mediated reduction in miR-221/222 expression and decreased the TNF-α-induced activation of p38 MAPK and NF-κB, thereby suppressing ICAM-1 expression and monocyte adhesion. Collectively, our results show that resveratrol attenuates endothelial inflammation by reducing ICAM-1 expression and that the protective effect was mediated partly through the miR-221/222/AMPK/p38/NF-κB pathway.

Proinflammatory cytokines, including tumor necrosis factor (TNF)-α, are thought to play an important role in vascular inflammation, leading to cardiovascular and metabolic disorders^1^. Endothelial cells (ECs) are responsible for cardiovascular homeostasis, and endothelial dysfunction has been shown to be involved in the pathophysiology of cardiovascular diseases^2^. Monocyte adhesion is a dynamic multistep process that includes the initial “rolling” of cells along the vessel endothelium in response to inflammatory mediators, arrest at the endothelium and subsequent strong adhesion to the systemic vasculature^3^. Accumulating evidence has demonstrated that a link between the upregulation of adhesion molecules such as intercellular adhesion molecule (ICAM)-1 on the surface of the ECs and the adhesion of circulating monocytes is pivotal in the development of inflammatory responses^4^. Several mechanisms have been indicated to regulate ICAM-1 expression in the endothelium, including the transcriptional and post-transcriptional regulation of the gene encoding ICAM-1, as well as post-transcriptional modifications. Post-transcriptional regulation mediated by micro RNAs, small RNA regulators, has also been suggested to participate in this process^5^. These molecules target mRNAs based on complementary sequences between the miRNAs and the 3′-untranslated regions (3′UTRs) of the target mRNAs, resulting in suppression of the target genes via the induction of mRNA degradation and/or translational suppression^6^. Because miRNAs appear to specifically regulate gene expression levels, rather than producing strict “on-off” decisions, they can be considered to fine-tune cellular responses to external influences^7^. Few studies have focused on the modulation of ICAM-1 expression by miRNAs. Examples of the miRNA-mediated regulation of ICAM-1 expression can be observed for miR-221/222, which have been shown to be associated with reduced ICAM-1 expression^8^. However, the role of these miRNAs in the context of TNF-α-treated ECs and monocyte adhesion has not yet been determined.

There is growing evidence that the disruption of cytokine-induced ICAM-1 expression exerts significant vasculoprotective action by attenuating vascular inflammation^9^. Therefore, identifying novel pharmacological methods to inhibit ICAM-1 expression holds great promise for the prevention of vascular inflammation and cardiovascular diseases. Resveratrol (trans-3,5,4′-trihydroxystilbene) is a natural phytoalexin found in grapes and red wine. A growing body of evidence indicates that resveratrol provides protection against numerous diseases, including cancer^10^, inflammation^11^ and cardiovascular diseases^12^. Resveratrol plays an important anti-inflammatory role in human umbilical vascular endothelial cells (HUVECs). Furthermore, the effects of resveratrol on the regulation of adhesion molecule expression involve a complex array of intracellular signaling pathways, including mitogen-activated protein kinases (MAPKs) and transcription factors^13^,^14^. Although these multiple signaling molecules have received considerable attention, the mechanisms underlying the resveratrol-mediated protection of ECs against adhesion molecule expression remain poorly understood, and a better understanding of these might provide important insights into the prevention of atherogenesis and inflammation. Therefore, we tested the ability of resveratrol to modulate the expression of adhesion molecules, MAPKs, transcription factors, and miR-221/222 in TNF-α-treated HUVECs. In addition, we examined the effects of resveratrol on ICAM-1 expression in TNF-α-treated wild-type (WT) and miR-221/222 knockout (KO) mice. Our results showed that resveratrol reduced ICAM-1 expression both *in vitro* and *in vivo* and that this effect was partly mediated by the inhibition of p38 phosphorylation and NF-κB activation. Resveratrol also significantly inhibited the adhesion of monocytes to TNF-α-treated HUVECs. These beneficial effects of resveratrol are involved in the miR-221/222-mediated protective process.

## Results

### Resveratrol reduces the TNF-α-induced expression of ICAM-1 mRNA and protein in HUVECs

**Figure 1:**
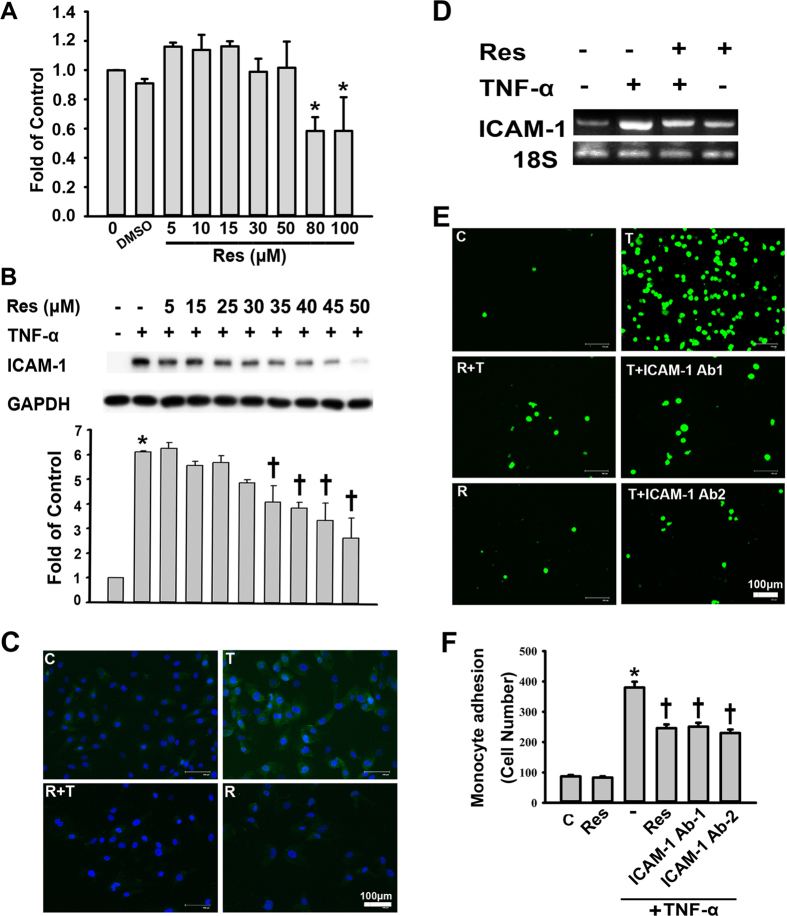
Resveratrol-mediated reduction in ICAM-1 mRNA and protein levels in TNF-α-treated HUVECs. (**A**) Treatment of HUVECs with different concentrations of resveratrol (Res); cell viability was assessed using the MTT assay. *P < 0.05 compared to untreated cells. (**B**) HUVECs were incubated with the indicated concentrations of resveratrol for 24 h and then with 3 ng/mL TNF-α for 4 h in the continued presence of resveratrol; ICAM-1 protein in cell lysates was then measured by Western blot. GAPDH was used as the loading control. The data are expressed as a fold value compared to the control value and are shown as the mean ± SEM for five separate experiments. Blots are cropped for clarity; full blots are shown in the Supplementary file. (**C**) HUVECs were incubated for 24 h with 50 μM of resveratrol (R); then, the cells were incubated with 3 ng/mL of TNF-α (T) for 4 h. ICAM-1 expression was analyzed by immunofluorescence staining. Bar = 100 μm. (**D**) Analysis of ICAM-1 mRNA levels in untreated HUVECs or HUVECs preincubated with or without 50 μM resveratrol for 24 h and then incubated with 3 ng/mL TNF-α for 4 h. Total RNA was analyzed by RT-PCR after normalization to 18S levels. (**E**) Representative fluorescence images showing the effects of resveratrol on the TNF-α-induced adhesion of fluorescein-labeled U937 cells to HUVECs. Cells were left untreated or were pretreated for 24 h with 50 μM resveratrol or for 1 h with 1 or 2 μg/mL anti-ICAM antibodies; then, they were treated with 3 ng/mL TNF-α for 4 h. BCECF-AM-labeled U937 cells were added to HUVECs and incubated at 37 °C for 45 min. The adherent cells were imaged by fluorescence microscopy. Bar = 100 μm. (**F**) The number of U937 cells bound per high power field in six randomly selected images was counted. The data are expressed as the mean ± SEM of three separate experiments. *P < 0.05 compared with the untreated cells. ^†^*P* < 0.05 compared with the TNF-α-treated cells.

Treatment of HUVECs with 3 ng/mL TNF-α for 24 h did not result in cytotoxicity, as determined by a 3-(4,5-dimethylthiazol-2-yl)-2,5-diphenyl tetrazolium bromide (MTT) assay (data not shown). After a 24-h incubation with 5, 10, 15, 30, 50, 80, or 100 μM of resveratrol, a significant reduction in cell viability was caused by the two highest concentrations (Fig. 1A). TNF-α (3 ng/mL) treatment for 4 h significantly induced ICAM-1 protein expression in HUVECs (Fig. 1B). Resveratrol significantly decreased ICAM-1 expression in a concentration-dependent manner; 35, 40, 45, or 50 μM of resveratrol caused a significant reduction in ICAM-1 expression. Based on these results, 3 ng/mL TNF-α and 50 μM resveratrol were used in the present study to evaluate the anti-inflammatory effects and molecular mechanisms of resveratrol treatment. Consistently, fluorescence microscopy images showed that ICAM-1 was strongly expressed in the cytosol of TNFα-treated HUVECs (Fig. 1C). In contrast, ICAM-1 expression was weaker in TNF-α-treated HUVECs with resveratrol pretreatment. To determine whether the effects of TNF-α alone or together with resveratrol on ICAM-1 expression were exerted at the transcriptional level, ICAM-1 mRNA levels were measured by RT-PCR. As shown in Fig. 1D, unstimulated HUVECs produced low levels of ICAM-1 mRNA, and 4-h treatment with TNF-α resulted in a significant increase in the ICAM-1 mRNA level. This increase was markedly inhibited by 24 h of preincubation with 50 μM resveratrol.

Since cell adhesion molecules are critical for monocyte adhesion, the next step was to examine the effect of resveratrol on monocyte adhesion. As shown in Fig. 1E and F, minimal monocyte binding to confluent control ECs was observed, but adhesion was substantially increased when the ECs were treated with TNF-α. In contrast, pretreating ECs with resveratrol significantly reduced the binding of the monocytes to TNF-α-treated ECs. In addition, the pretreatment of the ECs with a neutralizing antibody against ICAM-1 was used to explore the specificity of ICAM-1 in this process. After the pretreatment of ECs with 1 or 2 μg/mL ICAM-1 antibody, compared with non-antibody-incubated controls, the TNF-α-treated ECs exhibited a significantly reduced percentage of monocyte binding. In the presence of the ICAM-1 antibody, TNF-α failed to induce significant monocyte adhesion, thereby demonstrating the role of ICAM-1 in monocyte adhesion.

### The inhibition of p38 phosphorylation mediates the reduction in ICAM-1 expression by resveratrol in TNF-α-treated HUVECs

**Figure 2:**
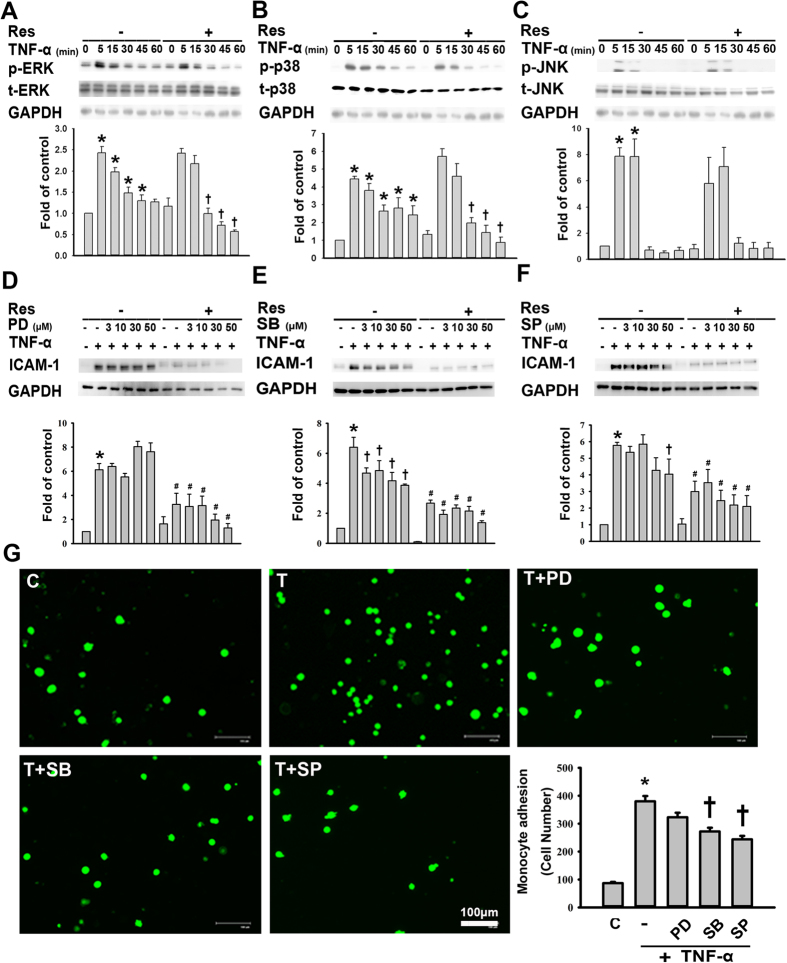
The resveratrol-mediated reduction in TNF-α-induced ICAM-1 expression is partly dependent on the inhibition of p38 phosphorylation. (**A**–**C**) The effects of resveratrol treatment on the phosphorylation of ERK1/2, p38, or JNK in TNF-α-treated HUVECs. HUVECs were incubated for 24 h with or without 50 μM resveratrol; then, the cells were incubated with 3 ng/mL of TNF-α for the indicated time, and aliquots of cell lysates containing equal amounts of protein subjected to immunoblotting with antibodies against (**A**) p-ERK1/2 and t-ERK1/2, (**B**) p-p38 and t-p38, (**C**) or p-JNK and t-JNK. (**D**–**F**) HUVECs were incubated for 23 h with or without 50 μM resveratrol, with or without the subsequent addition of the indicated concentrations of (**D**) PD98059 (an ERK1/2 inhibitor), (**E**) SB203580 (a p38 inhibitor), or (**F**) SP600125 (a JNK inhibitor) for 1 h in the continued presence of resveratrol. This was followed by incubation with or without TNF-α for 4 h, and then the cell lysates were analyzed for ICAM-1 expression by Western blot. The data are expressed as a fold of the control value and are shown as the mean ± SEM of three separate experiments. GAPDH was used as the loading control. Blots are cropped for clarity; full blots are shown in the Supplementary file. (**G**) Representative fluorescence images showing the effects of MAPK inhibitors on the TNF-α-induced adhesion of fluorescein-labeled U937 cells to HUVECs. Cells were left untreated or were pretreated for 1 h with PD98059 (30 μM), SB203580 (30 μM), or SP600125 (30 μM). Then, they were treated with 3 ng/mL TNF-α for 4 h in the continued presence of the inhibitor. BCECF-AM-labeled U937 cells were added to HUVECs and incubated at 37 °C for 45 min. The adherent cells were imaged by fluorescence microscopy. Bar = 100 μm. The number of U937 cells bound per high power field in six randomly selected images was counted. The data are expressed as the mean ± SEM of three separate experiments. *P < 0.05 compared with the untreated cells. ^†^*P* < 0.05 compared with the TNF-α-treated cells.

The contribution of the MAPK pathways to ICAM-1 expression in inflammatory cytokine-treated ECs has been previously reported^14^. We next investigated whether TNF-α-induced ICAM-1 expression was mediated by the activation of MAPKs. TNF-α treatment induced the transient phosphorylation of ERK1/2, p38, and JNK in HUVECs, with the maximal response occurring within 30 min, followed by a decline within 60 min (Fig. 2A). To determine the potential targets that were negatively regulated by resveratrol, the cells were preincubated with resveratrol for 24 h and then incubated with TNF-α for the indicated periods of time. Pretreatment with resveratrol significantly inhibited TNF-α-induced ERK and p38 phosphorylation at 30, 45, and 60 min (Fig. 2A,B), but pretreatment had no significant effect on JNK phosphorylation (Fig. 2C). In addition, pretreatment for 1 h with the indicated concentrations of SB203580 (a p38 inhibitor) or SP600125 (a JNK inhibitor) inhibited the TNF-α-induced ICAM-1 expression observed after 4 h of TNF-α treatment; however, PD98059 (an ERK1/2 inhibitor) had no significant effect (Fig. 2D–F). Importantly, HUVECs co-treated with resveratrol and any of the abovementioned inhibitors exhibited significantly reduced levels of TNF-α-induced ICAM-1 expression than did those treated with an inhibitor alone. These results suggest that resveratrol inhibits TNF-α-induced ICAM-1 expression partly by inhibiting TNF-α-induced p38 phosphorylation. As shown in Fig. 2H, inhibitors specific for MAPKs (SP600125 and SB203580) blocked TNF-α-induced monocyte adhesion, further supporting the idea that in HUVECs, the sequential activation of MAPK pathways results in the upregulation of ICAM-1 expression and subsequently in increased monocyte adhesion.

### Resveratrol decreases TNF-α-induced IκB phosphorylation and the phosphorylation, acetylation, and translocation of NF-κB p65 in HUVECs

**Figure 3:**
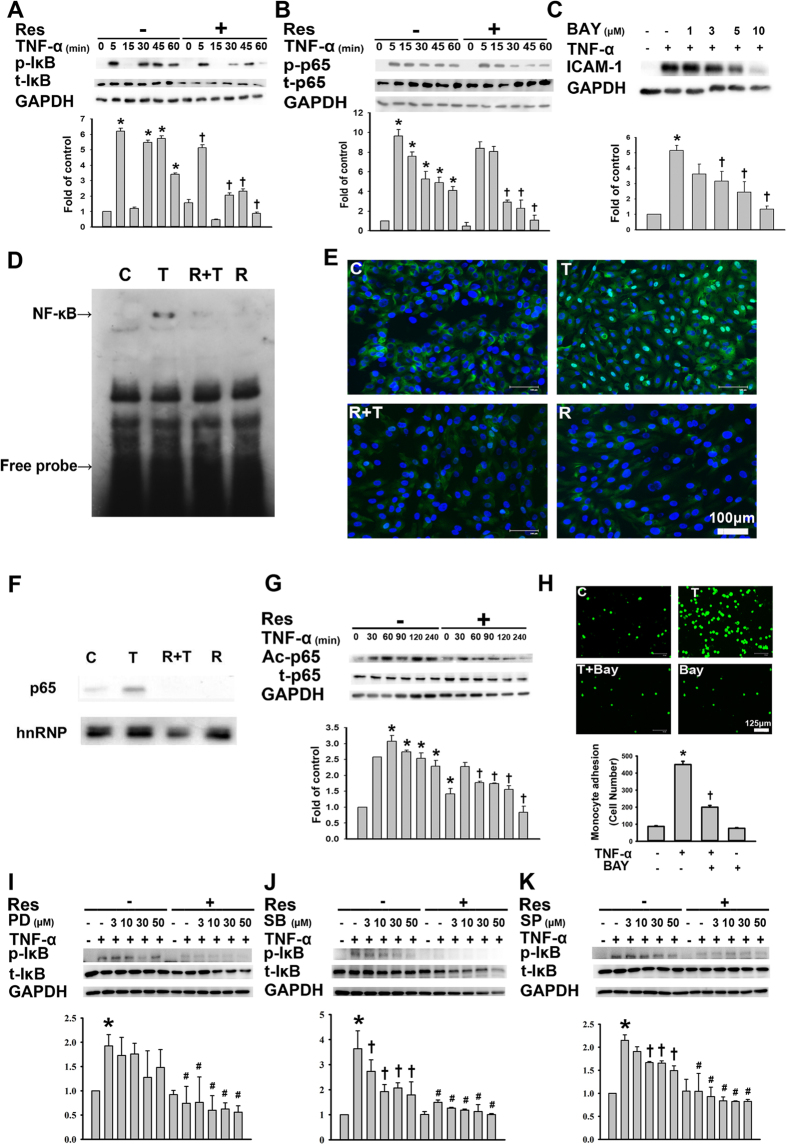
Resveratrol decreases TNF-α-induced IκB phosphorylation and the phosphorylation, acetylation, and translocation of NF-κB p65 in HUVECs. (**A**,**B**) Western blot analysis for the phosphorylation of IκB (**A**) and NF-κB p65 (**B**). HUVECs were preincubated for 24 h with 50 μM resveratrol and then were treated with 3 ng/mL TNF-α for the indicated time. (**C**) Cells were co-incubated for 24 h with 0–10 μM Bay117082 (an NF-κB inhibitor) and then with 3 ng/mL TNF-α. Cell lysates were prepared and assayed for ICAM-1 by Western blot. (**D**) Nuclear extracts were prepared from untreated cells or from cells with or without the 24 h pretreatment with 50 μM resveratrol and incubation with 3 ng/mL TNF-α for 1 h; these extracts were then tested for NF-κB DNA binding activity by EMSA. (**E**) Immunofluorescence staining for NF-κB p65. HUVECs were preincubated for 24 h with 50 μM resveratrol and were then treated with 3 ng/ml TNF-α for 4 h. Representative results from three separate experiments are shown. (**F**) Western blot for NF-κB p65 expression in nuclear extracts. (**G**) Western blot for the acetylation of NF-κB p65. HUVECs were preincubated for 24 h with 50 μM resveratrol and were then treated with 3 ng/mL TNF-α for the indicated time. The data are expressed as a fold of the control value and are shown as the mean ± SEM of three separate experiments. GAPDH and t-p65 were used as loading control. (**H**) The effect of Bay 117082 on U937 cells adhered to TNF-α-treated HUVECs. (**I**–**L**) HUVECs were incubated for 23 h with or without 50 μM resveratrol, and then with or without the indicated concentrations of PD98059 (**I**), SB203580 (**J**), or SP600125 (**K**) for 1 h in the continued presence of resveratrol. This was followed by incubation with or without TNF-α for 5 min, and then the cell lysates were analyzed for the phosphorylation of IκB using Western blot. The data are expressed as the means ± SEM for three separate experiments. *P < 0.05 compared with the untreated cells. ^†^*P* < 0.05 compared with the TNF-α-treated cells. Blots are cropped for clarity; full blots are shown in the Supplementary file.

We investigated whether resveratrol inhibited TNF-α-induced ICAM-1 expression via an effect on NF-κB because the ICAM-1 gene promoter contains consensus binding sites for this transcription factor^15^. Western blot analysis revealed higher levels of phosphorylated IκB, which is responsible for NF-κB activation^16^, and NF-κB p65 in TNF-α-stimulated HUVECs than in control cells; in addition, these results showed that resveratrol pretreatment significantly reduced the phosphorylation levels of IκB and p65 (Fig. 3A,B). Furthermore, pretreatment with increasing concentrations of Bay 117082, an NF-κB inhibitor, attenuated the TNF-α-induced upregulation of ICAM-1 expression (Fig. 3C). Consistent with the Western blot results, pretreatment with resveratrol for 24 h blocked the TNF-α-induced NF-κB binding activity, as demonstrated by an electrophoretic mobility-shift assay (EMSA) (Fig. 3D). To determine whether NF-κB activation was involved in the pretranslational effects of resveratrol on ICAM-1 expression, we examined the NF-κB p65 protein levels in the nuclei of TNF-α-treated HUVECs by immunofluorescence staining. HUVECs stimulated with TNF-α for 30 min showed marked NF-κB p65 staining in the nuclei, whereas resveratrol-pretreated cells showed weaker nuclear NF-κB p65 expression but stronger staining in the cytoplasm (Fig. 3E). A similar result was obtained by Western blotting analysis (Fig. 3F). Lysine 310 acetylation is critical for the full activation of the transcription regulation potential of NF-κB^17^. The acetylated-p65 levels were higher in TNF-α-treated HUVECs than in control HUVECs, and resveratrol pretreatment significantly reduced this effect (Fig. 3G). Moreover, we determined the role of the activated NF-κB pathway in TNF-α-mediated monocyte adhesion; cell pretreatment with Bay 117082 blocked TNF-α-induced monocyte adhesion (Fig. 3H), further lending support to the idea that in HUVECs, sequential NF-kB pathway activation leads to the upregulation of ICAM-1 expression and subsequent monocyte adhesion. To further elucidate the detailed pathway, we examined the crosstalk between MAPKs and NF-κB in HUVECs. HUVECs were pretreated with PD98059, SB203580, or SP600125 for 1 h and then stimulated with TNF-α for 5 min. As shown in Fig. 3I–K, all three inhibitors reduced the phosphorylation of IκB. Additionally, HUVECs co-treated with resveratrol and any of the abovementioned inhibitors exhibited significantly reduced IκB phosphorylation levels than did those treated with an inhibitor alone. These data suggested that the reduction in ICAM-1 expression caused by resveratrol in TNF-α-treated HUVECs was mediated by the p38/NF-κB signaling pathway.

### Resveratrol-mediated decreases in TNF-α-induced ICAM-1 expression are partly dependent on AMPK phosphorylation

**Figure 4:**
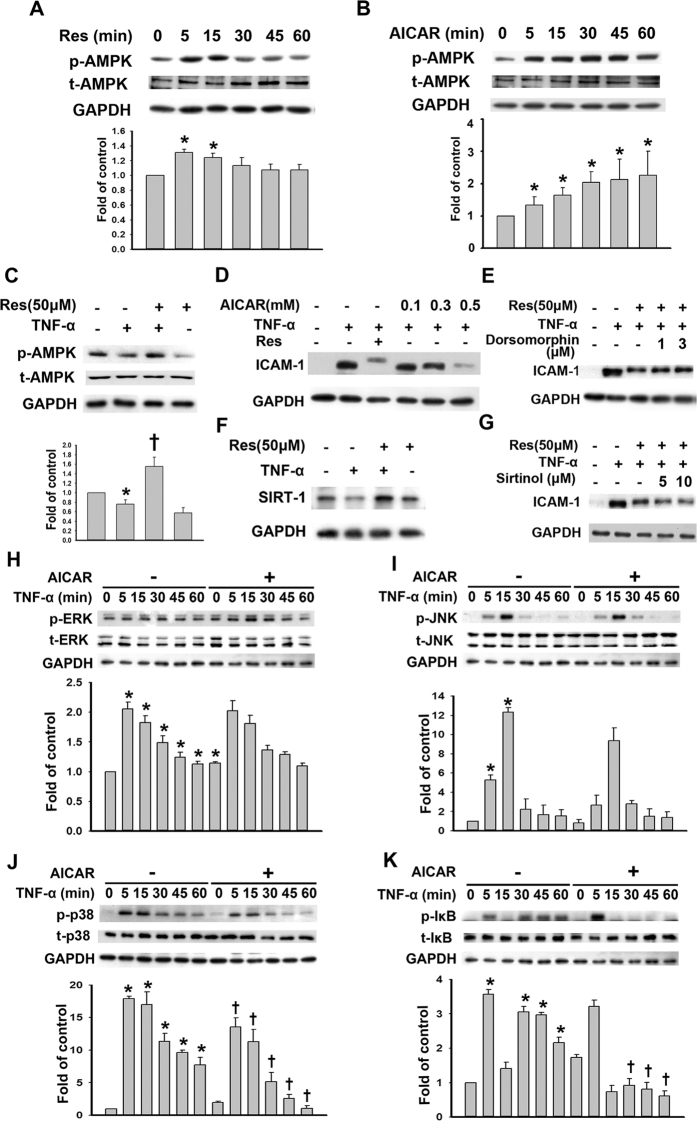
Resveratrol-mediated decreases in TNF-α-induced ICAM-1 expression and partly dependent on AMPK phosphorylation. (**A**) Representative immunoblot analysis of the time-dependence of the resveratrol-mediated phosphorylation of AMPK (p-AMPK) in HUVECs. The density of the p-AMPK bands was quantified and normalized to the protein loading control GAPDH, and the mean ± SEM are shown (n = 3). (**B**) Immunoblot analysis of the time-dependence of AICAR (an AMPK activator)-mediated AMPK phosphorylation. (**C**) Western blot analysis of the p-AMPK expression. HUVECs were preincubated for 24 h with 50 μM resveratrol and were then treated with 3 ng/mL TNF-α for 4 h. (**D**) Western blot analysis of ICAM-1 expression. HUVECs were incubated with the indicated concentrations of AICAR for 24 h and then with 3 ng/mL TNF-α for 4 h in the continued presence of AICAR; then, the ICAM-1 protein expression in cell lysates was measured by Western blot. (**E**) Western blot analysis of ICAM-1 expression. HUVECs were incubated with the indicated concentrations of Dorsomorphin (an AMPK inhibitor) and 50 μM resveratrol for 24 h and then with 3 ng/mL TNF-α for 4 h; then, the ICAM-1 protein expression in the cell lysates was measured by Western blot. (**F**) Western blot analysis of SIRT-1 expression. HUVECs were preincubated for 24 h with 50 μM resveratrol and were then treated with 3 ng/mL TNF-α for 4 h. (**G**) Western blot analysis of ICAM-1 expression. HUVECs were incubated with the indicated concentrations of Sirtinol (a SIRT-1 inhibitor) and 50 μM resveratrol for 24 h and then with 3 ng/mL TNF-α for 4 h; then, the ICAM-1 protein expression in the cell lysates was measured by Western blot. (H-K) The effects of AICAR treatment on p-ERK1/2, p-JNK, p-p38, and p-IκB in TNF-α-treated HUVECs. HUVECs were incubated for 24 h with or without 0.5 mM AICAR, and then the cells were incubated with 3 ng/mL of TNF-α for the indicated time. Cell lysates were subjected to immunoblotting with the antibodies against (**H**) p-ERK1/2 and t-ERK1/2, (**I**) p-p38 and t-p38, (**J**) p-JNK and t-JNK, or (**K**) p-IκB and t-IκB. *P < 0.05 compared with the untreated cells. ^†^*P* < 0.05 compared with the TNF-α-treated cells at the same time point. Blots cropped for clarity; full blots are shown in the Supplementary file.

As AMPK is a target of resveratrol in several model systems^18^,^19^,^20^, we investigated whether resveratrol could modulate the AMPK-mediated pathways that inhibit ICAM-1 expression. Compared with vehicle control-treated cells, significant AMPK activation was observed beginning at 5 min after resveratrol treatment and continuing for 15 min (Fig. 4A). Induced AMPK activity was also detected at 5 min after 5-aminoimidazole-4-carboxamide ribonucleoside (AICAR, 0.5 mM) administration, and the AMPK activity remained elevated during 1 h of AICAR exposure (Fig. 4B). Pretreatment with resveratrol significantly recovered the TNF-α-mediated reduction in AMPK phosphorylation (Fig. 4C). To investigate whether the inhibitory effect of resveratrol on ICAM-1 expression in TNF-α-treated HUVECs was due to AMPK activity, HUVECs were pretreated with the AMPK activator AICAR for 24 h. AICAR blocked the TNF-α-induced ICAM-1 expression in HUVECs in a dose-dependent manner (Fig. 4D). Dorsomorphin, an AMPK inhibitor, increased the reduced ICAM-1 expression caused by resveratrol in TNF-α-treated HUVECs (Fig. 4E). Pretreatment with resveratrol recovered the TNF-α-mediated reduction in SIRT-1 expression (Fig. 4F). However, the specific SIRT-1 inhibitor, Sirtinol, did not affect the resveratrol-reduced ICAM-1 expression in TNF-α-treated HUVECs (Fig. 4G). These results demonstrated that AMPK activation was involved in the inhibitory effect of resveratrol on TNF-α-induced ICAM-1 expression in HUVECs. To further elucidate the details of the pathway, we examined the crosstalk among AMPK, MAPKs and NF-κB in HUVECs. HUVECs were pretreated with AICAR for 1 h and then stimulated with TNF-α for the indicated periods of time. As shown in Fig. 4H–K, pretreatment with AICAR significantly inhibited TNF-α-induced p38 and IκB phosphorylation but had no significant effect on ERK or JNK phosphorylation. These data suggested that the reduction in ICAM-1 expression caused by resveratrol in TNF-α-treated HUVECs might be mediated by the AMPK/p38/NF-κB signaling pathway.

### Resveratrol-mediated reduction in ICAM-1 expression in TNF-α-treated HUVECs involves miR-221/222 upregulation

**Figure 5:**
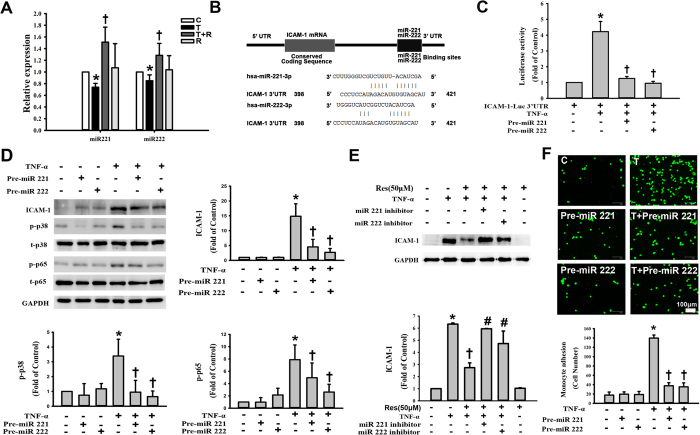
Resveratrol-mediated reduction in ICAM-1 expression in TNF-α-treated HUVECs involves miR-221/-222 upregulation. (**A**) Effect of resveratrol on miR-221/222 expression by RT-PCR in HUVECs after 4 h of exposure to TNF-α. RUN6B (U6) was used as the control. (**B**) The bioinformatics analysis of the potential target sites in the ICAM-1 3′ UTR for miR-221 or miR-222. (**C**) miR-221 and miR-222 significantly inhibit the TNF-α-induced ICAM-1 luciferase activity. HUVECs were co-transfected with the ICAM-1promoter plasmid and with miR-221 or miR-222 precursors for 48 h. The luciferase activity was measured in these cells after further incubated for 4 h with TNF-α. (**D**) Functional miR-221/222 overexpression decreased ICAM-1, p-p38, and p-p65 expression levels. HUVECs were transfected with miR-221 or miR-222 precursors for 48 h and then exposed to TNF-α for 4 h, followed by Western blot analysis for ICAM-1, p-p38, and p-p65 expression. (**E**) The effects of miR-221/222 inhibitors on resveratrol-reduced ICAM-1 expression in TNF-α-treated HUVECs. HUVECs were transfected with miR-221/222 inhibitors for 48 h and then treated with 50 μM resveratrol for 24 h and 3 ng/mL TNF-α for 4 h in the continued presence of miR-221/-222 inhibitors and resveratrol. ICAM-1 expression in the cell lysates was measured by Western blot. GAPDH was used as the loading control. Blots are cropped for clarity; full blots are shown in the Supplementary file. (**F**) Representative fluorescence images showing the effects of miR-221/222 overexpression on the adhesion of fluorescein-labeled U937 cells to TNF-α-treated HUVECs. The data are expressed as the mean ± SEM of three separate experiments. *P < 0.05 compared with the untreated cells. ^†^*P* < 0.05 compared with the TNF-α-treated cells.

Recent studies have suggested that ICAM-1 is a target of miR-221/-222, both of which regulate ICAM-1 expression in response to inflammatory stimuli^8^. To examine whether post-transcriptional regulation by miR-221/222 is critical for the TNF-α-induced expression of ICAM-1, HUVECs were treated with TNF-α and assessed for miR-221/-222 expression using RT-PCR. There was a significant decrease in the expression levels of both miR-221/-222 in HUVECs following TNF-α stimulation for 4 h, whereas resveratrol pretreatment increased miR-221/222 expression as assessed by real-time PCR (Fig. 5A). miR-221 and miR-222 are tandemly encoded on the X chromosome, are highly conserved and share significant homology. To test whether miR-221 and miR-222 target the ICAM-1 3′UTR, we used TargetScan.org (http://www.targetscan.org). We found that miR-221 and miR-222 are complementary to a region in the ICAM-1 3′UTR that is located between positions 413 and 419 (Fig. 5B). To confirm that miR-221/222 regulate the expression of ICAM-1, we constructed a firefly luciferase reporter plasmid containing the ICAM-1 3′UTR with miR-221/222 binding site. HUVECs were transfected with this luciferase gene reporter, and TNF-α treatment significantly increased the luciferase activity (Fig. 5C). In addition, HUVECs were co-transfected with this plasmid and either miR-221 or miR-222. The reporter assay showed that miR-221 and miR-222 could significantly inhibit the expression of the luciferase. These findings strongly suggested that TNF-α-induced ICAM-1 expression was mediated, at least in part, by miR-221/222 directly targeting 3′UTR of ICAM-1 in HUVECs.

Next, to test the role of miR-221/222 in the TNF-α-mediated induction of ICAM-1, HUVECs were transfected with miR-221/222 precursors for 48 h and then exposed to TNF-α for 4 h; subsequently, ICAM-1 protein expression was assessed by Western blot. As shown in Fig. 5D, the precursors of both miR-221/222 significantly attenuated TNF-α-induced ICAM-1 protein expression in HUVECs. However, the transfection of cells with a precursor control sequence did not inhibit the TNF-α-mediated induction of ICAM-1. A significant increase in the phosphorylation of p38 and p65 was detected in TNF-α-treated HUVECs, which was significantly attenuated in cells transfected with the miR-221/-222 precursors (Fig. 5D). HUVECs were also treated with sequence-specific inhibitors for miR-221/-222. The inhibitors reduced the resveratrol-mediated reduction in ICAM-1 expression in TNF-α-treated HUVECs (Fig. 5E). To determine the functional relevance of the miR-221/222-regulated expression of ICAM-1 in HUVECs, monocyte adhesion assays were performed using HUVECs transfected with miR-221/222 precursors. As shown in Fig. 5F, there was a significant reduction in monocyte adhesion to cells transfected with either of the precursors. These data further corroborate the hypothesis that the TNF-α-mediated suppression of miR-221/222 is involved in the expression of ICAM-1 in HUVECs and influences monocyte adhesion.

### Resveratrol reduces ICAM-1 expression in the ECs of thoracic aortas in TNF-α-treated WT mice but not miR-221/222 KO mice

**Figure 6:**
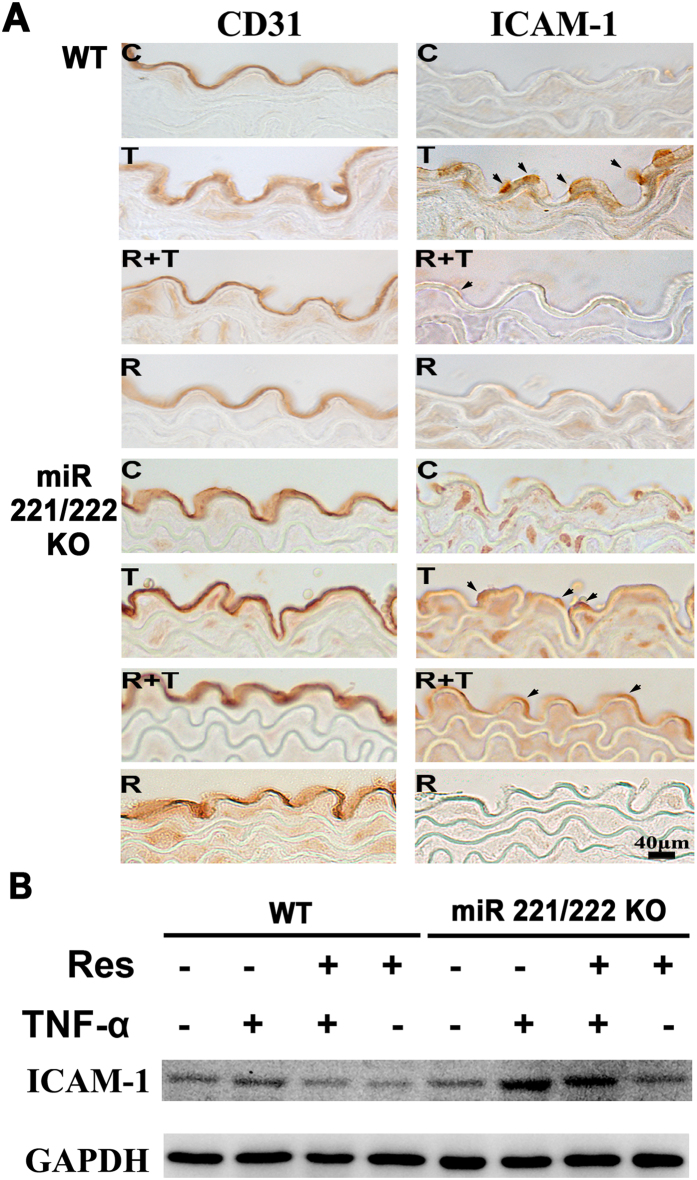
Resveratrol reduces ICAM-1 expression in the thoracic aorta ECs of TNF-α-treated WT mice but not miR-221/-222 KO mice. (**A**) Immunohistochemical staining for CD31 (an EC marker) and ICAM-1 expression in serial sections from the thoracic aortas of WT and miR-221/-222 KO mice. Mice were treated with DMSO, TNF-α, resveratrol + TNF-α, or resveratrol alone. The lumen is the uppermost portion in all sections. The reaction product is indicated by arrowheads. Bar = 40 μm. (**B**) Western blot analysis of ICAM-1 expression in aortic tissues of C57BL6J and miRNA-221/222 KO mice. Blots are cropped for clarity; full blots are shown in the Supplementary file.

To determine the effect of resveratrol on ICAM-1 expression *in vivo*, WT mice and miR-221/222 KO mice were injected with resveratrol for 3 days before being injected with TNF-α for 3 days. Then, immunohistochemical staining was performed to detect the expression of ICAM-1 on serial thoracic aorta sections, using CD31 as an EC marker. As shown in Fig. 6A, in the control and resveratrol-treated groups, no ICAM-1 staining was observed on the vascular wall, whereas in the TNF-α-treated group, strong ICAM-1 staining was observed on the luminal surface. In contrast, the preadministration of resveratrol resulted in weak ICAM-1 staining in the TNF-α-treated animals. ICAM-1 expression was strongly expressed in the TNF-α-treated miR-221/222 KO mice compared with the TNF-α-treated WT animals. Moreover, resveratrol did not decrease ICAM-1 expression in the miR-221/-222 KO mice. These results were similar to those of the Western blot analysis (Fig. 6B). The TNF-α treatment significantly induced ICAM-1 expression, whereas resveratrol significantly decreased ICAM-1 expression in aortic tissues with TNF-α treatment. TNF-α induced higher ICAM-1 expression in TNF-α-treated miR-221/-222 KO mice than in TNF-α-treated WT mice. ICAM-1 expression was also present with resveratrol treatment. These findings indicate that the beneficial effects of resveratrol were lost in miR-221/222 KO mice.

## Discussion

Here, we demonstrated that resveratrol treatment significantly attenuated ICAM-1 mRNA and protein expression and monocyte adhesion in TNF-α-treated HUVECs. This influence was partly mediated through the inhibition of p38 phosphorylation and NF-κB activation, as well as the activation of AMPK. Furthermore, miR-221/222 expression was markedly downregulated in TNF-α-treated HUVECs, whereas resveratrol upregulated the expression of miR-221/-222. Moreover, resveratrol reduced ICAM-1 expression in the thoracic aorta ECs of TNF-α-treated WT mice but not in those of miR-221/222 KO mice. The protection of ECs by resveratrol against inflammation is due to the inhibition of p38/NF-κB and ICAM-1 by miR-221/222.

Resveratrol, a naturally occurring phytoalexin with anti-inflammatory properties, can attenuate endothelial dysfunction, an initial step in cardiovascular progression^21^,^22^. Resveratrol was reported to attenuate ICAM-1 mRNA expression in TNF-α-treated coronary arterial ECs^23^. Resveratrol inhibited TNF-α-induced ICAM-1 and VCAM-1 expression in baboon femoral arterial ECs, as measured by flow cytometry^24^. In line with these reports, we demonstrated that resveratrol strongly reduced the ICAM-1 expression in TNF-α-treated ECs, as determined by Western blot, immunofluorescence staining and RT-PCR *in vitro*. Moreover, resveratrol decreased the ICAM-1 expression of thoracic aortas ECs in TNF-α-treated mice *in vivo*. Furthermore, resveratrol treatment markedly inhibited leukocyte adhesion to HUVECs by inhibiting ICAM-1 expression. ICAM-1 is considered a vital adhesion molecules for leukocyte recruitment to inflamed areas^25^, indicating that resveratrol treatment may have an important effect in preventing the progression of inflammation and cardiovascular diseases.

MAPK pathways play key roles in the regulation of cell adhesion molecules expressed on ECs in response to external stimuli^26^. The present study demonstrated that TNF-α caused the strong activation of three MAPK subtypes in ECs, as reported in the previous study^14^. Our results further showed that resveratrol decreased TNF-α-induced ERK1/2 and p38 phosphorylation. The increase in ICAM-1 expression induced by TNF-α was markedly suppressed in the presence of a p38 or JNK inhibitor, but not an ERK1/2 inhibitor. Based on these results, we suggest that one of the signals by which resveratrol attenuates TNF-α-induced ICAM-1 expression in ECs involves a reduction in p38 activation. Consistent with our results, the level of p38 phosphorylation was downregulated by resveratrol, which has been shown to ameliorate the high-glucose-induced upregulation of ICAM-1^27^ and diabetes-induced cardiac dysfunction^28^, as well as interleukin-6 release in PC12 cells^29^. Furthermore, increasing evidence shows that MAPK signaling pathways are involved in the regulation of NF-κB activation in TNF-α-treated ECs^30^. Our results demonstrated that the activation of NF-κB is necessary for the induction of ICAM-1 expression by TNF-α in HUVECs. We further demonstrated that the attenuation of TNF-α-induced ICAM-1 expression by resveratrol was mediated through the inhibition of IκB phosphorylation and the phosphorylation, activation, acetylation, and translocation of NF-κB p65.

Both the AMPK and SIRT1 pathways have been shown to play crucial roles in the inactivation of inflammatory mediators, inflammatory cell recruitment and immune cell functions^31^. These findings were confirmed by our observation that TNF-α treatment inhibited the phosphorylation of AMPK and SIRT1. In addition, the increase in ICAM-1 expression induced by TNF-α was markedly suppressed in the presence of AICAR, an AMPK activator. Furthermore, resveratrol treatment reversed the reduction in AMPK phosphorylation and SIRT1 expression caused by TNF-α. Resveratrol had a pronounced effect on AMPK phosphorylation and subsequently reduced ICAM-1 expression. Thus, one of the mechanisms by which resveratrol reduces TNF-α-induced ICAM-1 expression involves an increase in AMPK activation. This is comparable with the findings of a recent study indicating that resveratrol reversed AMPK deactivation and reduced ICAM-1 expression in diabetic retinas^18^. Moreover, there are multiple crosstalk points among AMPK and MAPKs pathways, the coordinated action of which determines the cell fate^32^. In our study, neither the phosphorylation of ERK or JNK was affected by the AMPK activator, but the phosphorylation of IκB and p38 was inhibited by the AMPK activator. Together, these results suggest that resveratrol treatment reverses the TNF-α-mediated reduction in AMPK phosphorylation, which in turn reduces the phosphorylation of the p38/NFκB/IκB cascades, and subsequently suppresses ICAM-1 expression, resulting in decreased leukocyte binding.

Recent reports indicate that ICAM-1 expression is controlled by many miRNA species, such as miR-221 and miR-222^33^,^34^. These miRNAs are complementary to the ICAM-1 3′UTR region and modulate ICAM-1 expression at the post-transcriptional level by binding to the UTR^5^. A previous study demonstrated that increased ICAM-1 expression in human immunodeficiency virus-1 Tat-treated ECs was concomitant with a reduction of miR-221/222 expression^8^. Interferon-γ suppressed miR-221, resulting in increased ICAM-1 expression in cholangiocytes^33^. In the present study, for the first time, we report the role of TNF-α in modulating the regulation of ICAM-1 via miR-221/222 in HUVECs. Importantly, we demonstrated that resveratrol treatment increased miR-221/222 expression. Additional results confirmed that the transfection of miR-221/222 precursors significantly decreased TNF-α-induced ICAM-1 expression and monocyte adhesion. Furthermore, we also demonstrated that miR-221/222 overexpression involves the inactivation of p38 and NF-kB-p65 in TNF-α-treated ECs. Further validation of these *in vitro* findings was carried out *in vivo* using a well-established miR-221/-222 KO mouse model. The immunohistochemical staining of mouse aorta samples revealed a notable increase in ICAM-1 expression specific to the aortic endothelial layer of the TNF-α-treated-miR-221/222 KO mice, whereas resveratrol treatment had no effect on TNF-α-induced ICAM-1 expression in these mice. Altogether, these findings demonstrate the important role of miR-221/222 in the resveratrol-mediated reduction in ICAM-1 expression and monocyte adhesion in TNF-α-treated ECs.

In summary, this study provides the first evidence that the anti-inflammatory effects of resveratrol are mediated through increased miR-221/222 expression and blockade NF-κB and p38 phosphorylation, resulting in decreased ICAM-1 expression and monocyte adhesion to ECs under inflammatory conditions. Hence, resveratrol may offer the novel therapeutic option for targeting cardiovascular disorders and inflammation.

## Materials and Methods

### Cell culture

Primary cultures of HUVECs were prepared as previously described^35^. The cells were grown in medium 199 (Gibco, NY, USA) containing 1% penicillin/streptomycin, 30 μg/mL of EC growth supplement (R&D Systems, Minneapolis, MN), and 10% fetal bovine serum (Biological Industries, Israel) at 37 °C in a humidified atmosphere of 95% air/5% CO_2_. Cells between passages 1 and 3 were used for experiments.

### MTT Cell viability assay

The MTT assay was used to measure cell viability^36^. The principle of this assay is that mitochondrial dehydrogenase in viable cells reduces MTT to a blue formazan. Briefly, cells were grown in 48-well plates and incubated with various concentrations of resveratrol for 24 h or TNF-α for 4 h; then, 100 μL of MTT (0.5 mg/mL) was added to each well and incubated at 37 °C for an additional 30 min. The medium was then carefully removed to avoid disturbing the formazan crystals that had formed. Dimethyl sulfoxide (DMSO; 150 μL), which solubilizes formazan crystals, was added to each well and the absorbance of the solubilized blue formazan was read at 530 nm (reaction) and 690 nm (background) using a DIAS Microplate Reader (Dynex Technologies, USA). The reduction in optical density caused by TNF-α and resveratrol was used as a measurement of cell viability and normalized to that of cells incubated in control medium, which were considered 100% viable.

### Reverse transcription and RT-PCR

Total RNA was extracted using TRIzol reagent (Invitrogen), according to the manufacturer’s protocol. The reverse transcriptase reaction was performed using M-MLV reverse transcriptase (Invitrogen). Complementary DNA was generated by the addition of 1 μg of total RNA to a reaction mixture containing 0.5 μg/μL oligo-deoxythymidine, 20 mM dNTP, 0.1 M dithiothreitol, 250 mM Tris-HCl, pH 8.3, 375 mM KCl, and 15 mM MgCl_2_ and allowing reaction at 37 °C for 90 min. The oligonucleotide primers used were 5′-CCGGAAGGTGTGAACTG-3′ (forward) and 5′-CTCCTCTCTAGTGGTACCT-3′ (reverse) for ICAM-1 and 5′-GTAACCCGTTGAACCCCATT-3′ (forward) and 5′-CCATCCAATCGGTAGTAGCG-3′ (reverse) for 18 S subunit ribosomal RNA. The amplification profile was 1 cycle of initial denaturation at 94 °C for 5 min and 30 cycles of denaturation at 94 °C for 1 min, primer annealing at 62 °C for 1 min, and extension at 72 °C for 5 min. The PCR products were analyzed on ethidium bromide -stained 2% agarose gels. To analyze miR-221/222 expression, comparative real-time PCR was performed by using the TaqMan microRNA Reverse Transcription kits. (Applied Biosystems, Foster City, CA, USA). TaqMan microRNA assay kits for miR-221 (000524), miR-222 (002276) and RNU6B (001973) were obtained from Applied Biosystems. All reactions were run in triplicate. The amounts of miR-221/222 were obtained by normalizing them to that of RNU6B and the control.

### Transfection of miR-221/222

To manipulate the cellular functions of miR-221/222 in HUVECs, we transfected cells with miR-221/222 precursors to increase miR-221/222 expression levels or with miR-221/222 inhibitors to decrease miR-221/222 expression levels. Briefly, HUVECs were grown to 70% confluence and transfected with the miR-221/222 mimic or miR-221/222 inhibitor (Dharmacon, CO, USA) at a concentration of 100 nM/well using Lipofectamine 3000 (Invitrogen, CA, USA), followed by an analysis of ICAM-1 expression and cell adhesion.

### Luciferase reporter assay

HUVECs were transfected with 1 μg of the ICAM-1 luciferase reporter construct for 24 h. These cells were co-transfected with or without 100 nM of premiR-221 or premiR-222. After 24 h of transfection, the cells were incubated with TNF-α for 4 h and were then harvested. Luciferase activity was measured using a Reporter Assay Kit (Promega Corp., Madison, WI, USA).

### Preparation of cell lysates and Western blot analysis

To prepare cell lysates, the cells were lysed for 1 h at 4 °C in 20 mM Tris-HCl, 150 mM NaCl, 1 mM EDTA, 1 mM EGTA, 1% Triton X-100, and 1 mM phenylmethylsulfonyl fluoride, pH 7.4; then, the lysates were centrifuged at 4000 *g* for 15 min at 4 °C, and the supernatants were retained. Cell lysate samples (20 μg of protein) were subjected to 10% sodium dodecyl sulfate-polyacrylamide gel electrophoresis and transferred to polyvinylidene fluoride membranes (Pall Corporation, NY, USA), which were then incubated for 30 min at room temperature with 5% nonfat milk in Tris-buffered saline containing 0.2% Tween 20 (TBST) to block the nonspecific binding of antibodies. All antibody dilutions were performed using TBST. The membranes were then incubated overnight at 4 °C with rabbit antibodies against human ICAM-1 (Santa Cruz; 1:10000 dilution), human phospho-JNK, human-JNK (Cell Signaling; 1:1000 dilution), human phospho-ERK1/2, human-ERK1/2 (Cell Signaling; 1:10000 dilution), human phospho-AMPK, human AMPK (Cell Signaling; 1:3000 dilution), human phospho-p38, human p38 (Santa Cruz Biotechnology; 1:5000 dilution), human p-65 or phospho-p65 (both from Abcam; 1:1000 dilution), human acetyl-Lys310-p65 (Biotech), or human SIRT1 (Proteintech Group; 1:2000 dilution), and then incubated for 1 h at room temperature with horseradish peroxidase (HRP)-conjugated goat anti-rabbit IgG antibodies (Santa Cruz; 1:5000 dilution). The bound antibodies were detected using Chemiluminescence Reagent Plus (NEN, MA, USA), and the intensity of each band was quantified using a densitometer. Antibodies against GAPDH (Santa Cruz Biotechnology; 1:10000 dilution) were used as loading controls.

### Immunocytochemical localization of ICAM-1 and NF-κB p65

To determine the localization of ICAM-1 or NF-κB p65 expression *in situ*, confluent HUVECs (controls or cells treated for 24 h with resveratrol) on slides were incubated alone or in the presence of 3 ng/mL TNF-α for 30 min, fixed in 4% paraformaldehyde in pH 7.4 PBS for 15 min at 4 °C, and incubated for 1 h at room temperature with rabbit anti-human ICAM-1 or NF-κB p65 antibodies (Cell Signaling; 1:50 dilution in PBS-NGS). After being washed, the slides were incubated for 1 h at 37 °C with fluorescein isothiocyanate-conjugated goat anti-rabbit IgG antibodies (Epitomics; 1:100 dilution in PBS-NGS) and viewed by fluorescence microscopy.

### EMSA

The preparation of nuclear protein extracts and the EMSA procedure have been previously described^37^. Nuclear proteins were extracted using NE-PER reagent (Pierce, Rockford, IL, USA) according to the manufacturer’s protocol. The NF-κB binding activity of equal amount (10 μg) of nuclear protein was analyzed using a LightShift Chemiluminescence EMSA kit (Pierce). The synthetic double-stranded oligonucleotides used as the probes in the gel-shift assay were 5′-AGTTGAGGGGACTTTCCCAGGC-3′ and 3′-TCAACTCCCCTGAAAGGGTCCG-5′ for NF-κB.

### Cell adhesion assay

HUVECs were left untreated or were pretreated for 24 h with 50 μM resveratrol; for 1 h with 1 or 2 μg/mL anti-ICAM-1 antibodies; or for 1 h with PD98059 (30 μM), SP600125 (30 μM), SB203580 (30 μM), or Bay117082 (10 μM). Then, they were treated with 3 ng/mL TNF-α for 4 h in the continued presence of the inhibitor. U937 cells, originally derived from a human histiocytic lymphoma and obtained from the American Type Culture Collection (Rockville, MD, USA), were labeled with 10 mM of BCECF/AM (Boehringer Mannheim, Mannheim, Germany) for 1 h at 37 °C and were then suspended in the same medium used for culturing HUVECs. The labeled U937 cells were washed and incubated with HUVECs in a 48-well plate for 1 h. The medium containing non-attached U937 cells was removed, and the cells remaining in the wells were washed twice with PBS. The number of U937 cells adhering to HUVECs was counted in six randomly selected images captured by a fluorescence microscope for each experiment.

### Mouse model and immunohistochemical staining

C57BL/6 WT mice and miR-221/-222 KO mice (weight: 25–30 gm; age: 9–12 weeks) were used in this study. We generated miR-221/-222 KO mice by deleting the X-linked miR-221/222 genes and breeding the mice for 10 generations on a C57BL/6 background (unpublished result). These mice were viable and fertile. All mice were maintained in the Laboratory Animal Center of the Department of Bioscience Technology of Chung Yuan Christian University. All animal procedures were approved by the Animal Ethics Committee of National Taiwan University (NTU-20140119) and Chung Yuan Christian University (CYCU-10123). All procedures involving experimental animals were performed in accordance with the guidelines for animal care of National Taiwan University and Chung Yuan Christian University and complied with the *Guide for the Care and Use of Laboratory Animals*, NIH publication No. 86–23, revised 1985. The mice were randomly divided into four groups, which were to be treated with DMSO, TNF-α, TNF-α plus resveratrol, or resveratrol only. The mice were orally administered resveratrol (10 mg/Kg/day in 50 μL of DMSO) or DMSO (50 μL) for 5 days and were then left untreated or were injected intraperitoneally with TNF-α (10 μg/Kg/day) for the next 3 days. They were then anesthetized by the intraperitoneal injection of 30–40 mg/Kg pentobarbital and sacrificed; then, the thoracic aortas were excised, immersion-fixed in 4% buffered paraformaldehyde, paraffin-embedded, and cross-sectioned for immunohistochemistry. To determine the level of ICAM-1 expression in the aortic walls and whether it was associated with ECs, two serial sections were examined by immunostaining for CD31 (an EC marker) or ICAM-1. The first section was incubated sequentially for 1 h at 37 °C with mouse monoclonal anti-human CD31 antibody (1:50 dilution; Neomarkers, CA, USA) and 1 h at room temperature with HRP-conjugated goat anti-mouse IgG antibodies (1:200 dilution; Sigma) and bound antibody were visualized using 3,3′-diaminobenzidine (Sigma-Aldrich). The second section was incubated with rabbit antibodies against human ICAM-1 (1:100; Santa Cruz Biotechnology) at 4 °C for 1 h, washed with PBS, and then incubated with an HRP-conjugated second antibody and the same chromogen described above.

### Statistical analysis

All data are expressed as the mean ± SEM. Differences in the mean values among different groups were analyzed by one-way ANOVA and a post hoc Dunnett test. A value of *P* < 0.05 was regarded as statistically significant.

## Additional Information

**How to cite this article:** Liu, C.-W. *et al*. Resveratrol attenuates ICAM-1 expression and monocyte adhesiveness to TNF-α-treated endothelial cells: evidence for an anti-inflammatory cascade mediated by miR-221/222/AMPK/p38/NF-κB pathway. *Sci. Rep.* **7**, 44689; doi: 10.1038/srep44689 (2017).

**Publisher's note:** Springer Nature remains neutral with regard to jurisdictional claims in published maps and institutional affiliations.

## Supplementary Material

Supplementary Information
