# Supplementary material for: Resveratrol attenuates ICAM-1 expression and monocyte adhesiveness to TNF-α-treated endothelial cells: evidence for an anti-inflammatory cascade mediated by the miR-221/222/AMPK/p38/NF-κB pathway
Source: Sci Rep. 2017 Mar 24;7:44689. doi: 10.1038/srep44689 (PMC5364502; doi:10.1038/srep44689)
Supplement: Supplementary Information [file srep44689-s1.doc]

Supplementary Information

**Title:**

Resveratrol attenuates ICAM-1 expression and monocyte adhesiveness to TNF-α-treated endothelial cells: evidence for an anti-inflammatory cascade mediated by miR-221/-222/AMPK/p38/NF-κB pathway

**Authors:**

Chen-Wei Liu, Hsin-Ching Sung, Shu-Rung Lin, Chun-Wei Wu, Chiang-Wen Lee, I-Ta Lee, Yi-Fan Yang, I-Shing Yu, Shu-Wha Lin, Ming-Hsien Chiang, Chan-Jung Liang, Yuh-Lien Chen


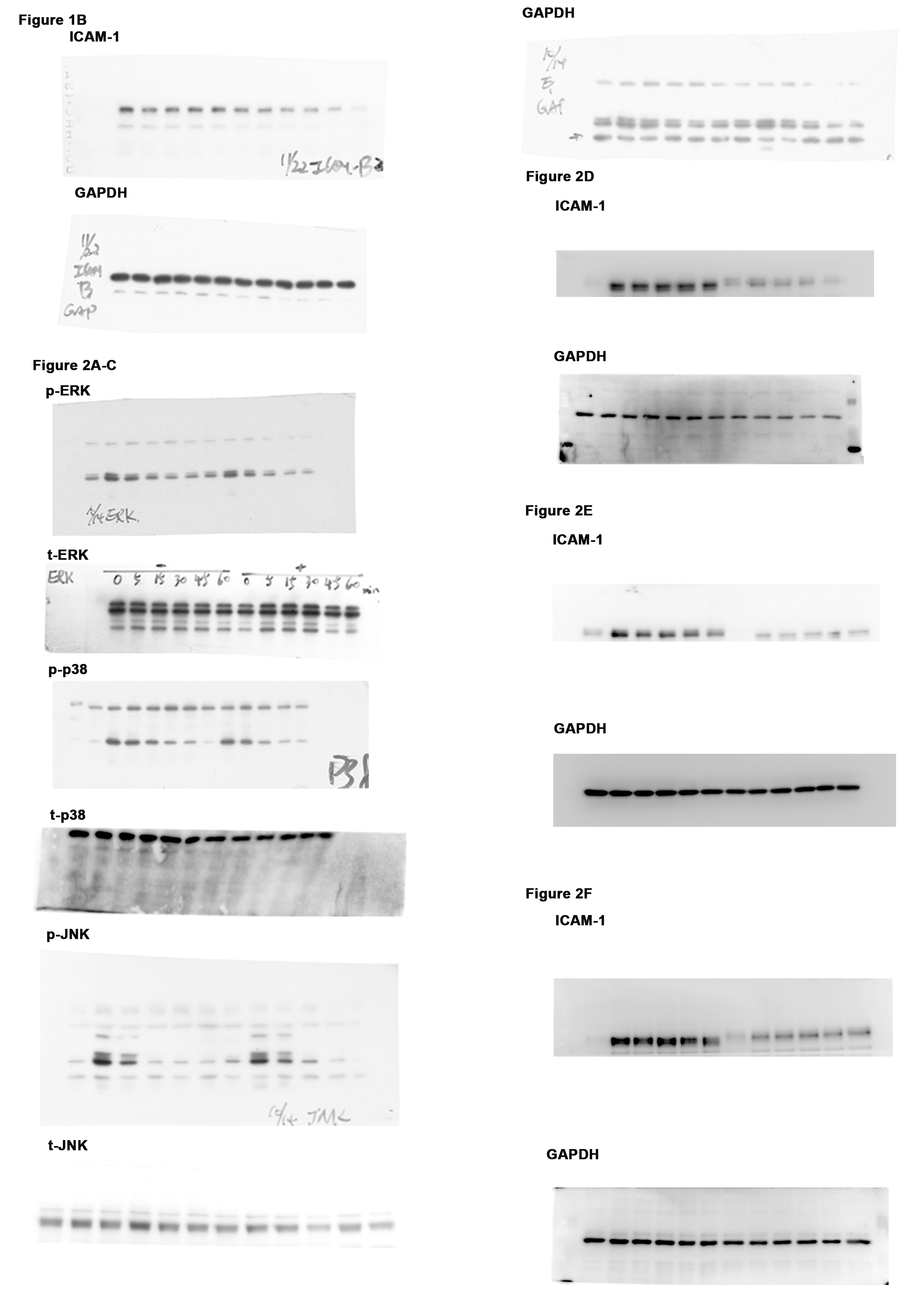


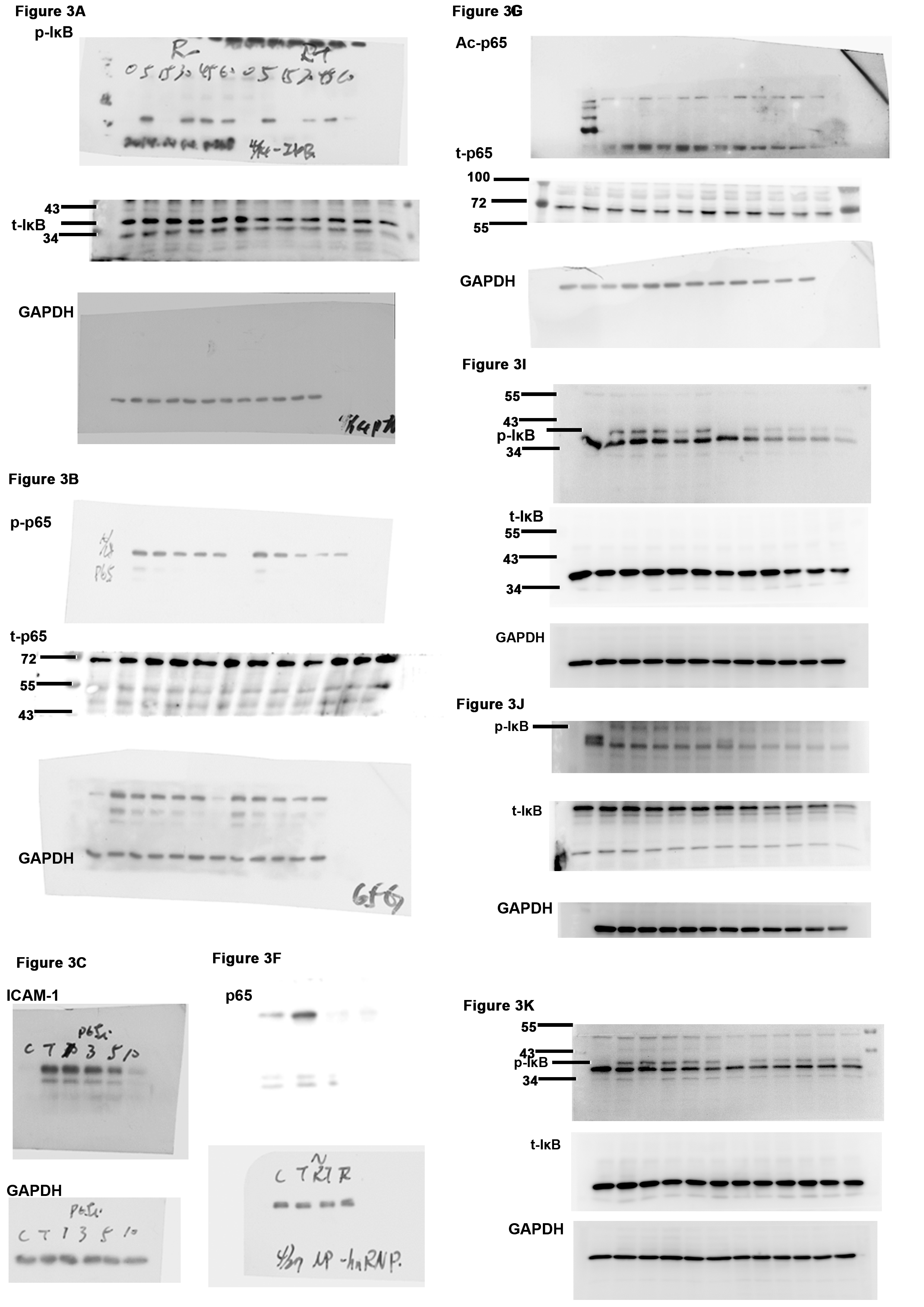


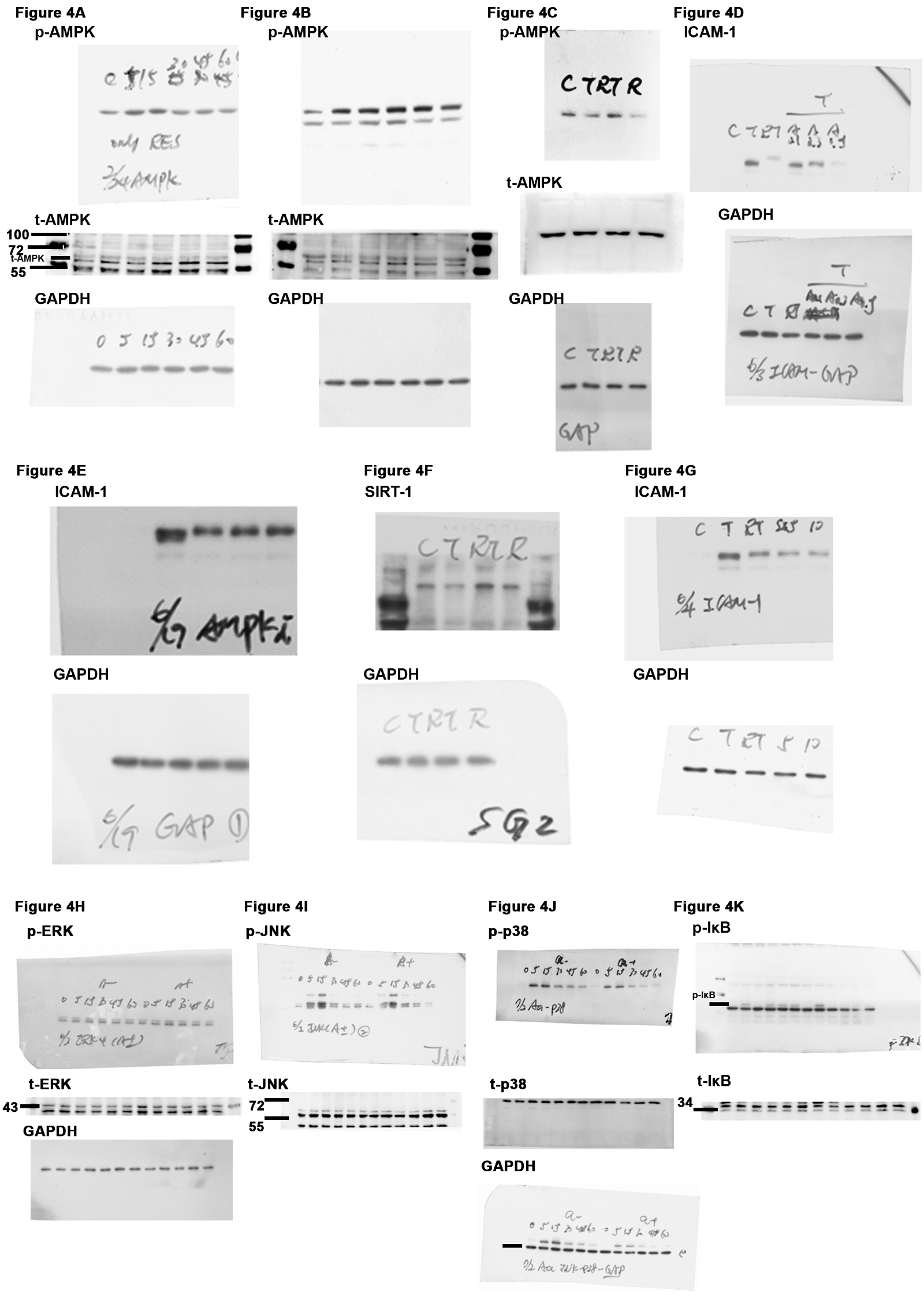


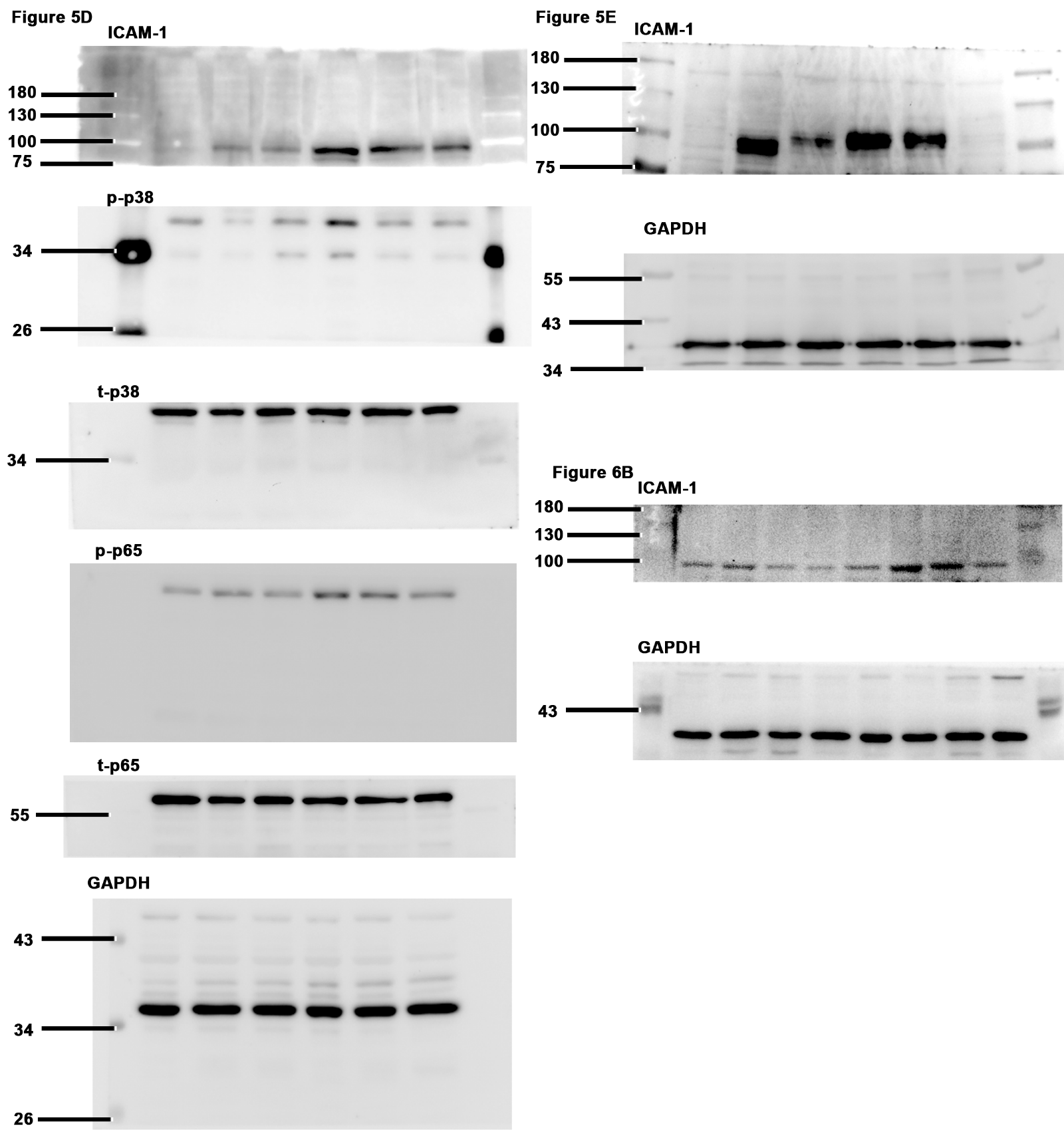


Figure S1. Full-length blots for the indicated figures from the main text.
